# An Evolutionarily Threat-Relevant Odor Strengthens Human Fear Memory

**DOI:** 10.3389/fnins.2020.00255

**Published:** 2020-04-22

**Authors:** Jessica E. Taylor, Hakwan Lau, Ben Seymour, Aya Nakae, Hidenobu Sumioka, Mitsuo Kawato, Ai Koizumi

**Affiliations:** ^1^Department of Decoded Neurofeedback (DecNef), Computational Neuroscience Laboratories, Advanced Telecommunications Research Institute International, Kyoto, Japan; ^2^Department of Psychology, Brain Research Institute, University of California, Los Angeles, Los Angeles, CA, United States; ^3^Department of Psychology, University of Hong Kong, Pokfulam, Hong Kong; ^4^Center for Information and Neural Networks (CiNet), National Institute of Information and Communications Technology (NICT), Osaka, Japan; ^5^Computational and Biological Learning Lab, Department of Engineering, University of Cambridge, Cambridge, United Kingdom; ^6^Department of Neural Computation for Decision-Making, Cognitive Mechanisms Laboratories, Advanced Telecommunications Research Institute International, Kyoto, Japan; ^7^Graduate School of Frontier Biosciences, Osaka University, Osaka, Japan; ^8^Hiroshi Ishiguro Laboratories, Advanced Telecommunications Research Institute International, Kyoto, Japan; ^9^Sony Computer Science Laboratories, Inc., Tokyo, Japan

**Keywords:** fear memory, human olfaction, predator odor, innate fear, contextual memory modulation

## Abstract

Olfaction is an evolutionary ancient sense, but it remains unclear to what extent it can influence routine human behavior. We examined whether a threat-relevant predator odor (2-methyl-2-thiazoline) would contextually enhance the formation of human fear memory associations. Participants who learned to associate visual stimuli with electric shock in this predator odor context later showed stronger fear responses to the visual stimuli than participants who learned in an aversiveness-matched control odor context. This effect generalized to testing in another odor context, even after extinction training. Results of a separate experiment indicate that a possible biological mechanism for this effect may be increased cortisol levels in a predator odor context. These results suggest that innate olfactory processes can play an important role in human fear learning. Modulatory influences of odor contexts may partly explain the sometimes maladaptive persistence of human fear memory, e.g., in post-traumatic stress disorders.

## Introduction

Although odors can act as powerful cues for triggering *recall* of fear memories (Vermetten and Bremner, 2003; Hinton et al., 2004), their ability to act as contextual modulators of fear memory *formation* is less well understood. Many previous studies have exposed experimental animals to odors at the time of fear memory formation; however, these studies typically used odors as a direct cue for the learned association. Often, in these experiments, neutral odors were used as conditioned stimuli (CS) (e.g., Richardson et al., 1999; Paschall and Davis, 2002; Sevelinges et al., 2004; Jones et al., 2005; Kass et al., 2013; Shionoya et al., 2013) and/or aversive odors were used as unconditioned stimuli (US) (e.g., Blanchard et al., 2001, 2003; Endres and Fendt, 2007). The number of studies exposing human subjects to odors at the time of fear or negative memory formation is far fewer, but these have also tended to use odors as a direct cue for the learned association (either as a CS, e.g., Kirk-Smith et al., 1983; Epple and Herz, 1999; Parma et al., 2015; or as a US, e.g., Schneider et al., 1999; Hermann et al., 2002). Therefore, despite suggestions that their pervasiveness might cause odors to provide particularly strong contextual information (Herz and Engen, 2010), there remains little known about how an odor might affect fear learning when it serves (neither as CS or US but) simply as a background context. Better elucidation of modulatory contextual features could be beneficial for the understanding of why some, but not all, humans who experience trauma develop robust fear memories that potentially lead to fear-related disorders.

In this study, we hypothesized that an odor providing evolutionary threat-relevant contextual information may influence human fear memory formation. This is because humans display threat-related psychological responses in evolutionary threat-relevant odor contexts (Stevenson, 2010; Wisman and Shrira, 2015), indicating that we are capable of processing their threat relevance. For example, putrescine (the smell of decay) has been shown to elicit human threat management mechanisms (Wisman and Shrira, 2015), and the body odor of scared humans has been shown to influence the cognitive performance of other humans (Chen et al., 2006). A review of the animal literature shows that a simple context containing a predator odor is often sufficient to induce evolutionary fear-like responses (Apfelbach et al., 2005). The mechanism behind the effect of a predator odor is thought to be innate (Kobayakawa et al., 2007) and has been found to exceed the effect of an experimentally fear-conditioned threat odor on appetitive behavior (Isosaka et al., 2015). The molecular and neural mechanisms behind innate fear-like responses driven by predator odors are relatively well known (Kobayakawa et al., 2007; Isosaka et al., 2015; Wang et al., 2018). If predator odors affect humans innately as well, then this opens the scope for a broad range of future research. Therefore, in this study, we decided to specifically test the effect of a predator odor.

Predator odors may not typically be thought to be innately threat-relevant to humans, but they have previously been suggested to contain chemicals that have been reliable indicators of predators in our ancestral past (Stevenson, 2010). Our fear circuits have evolved over history so that we potentially retain the innate capacity to process certain stimuli from our evolutionary past as threatening, even if they no longer signify real danger to us (LeDoux, 2019). Indeed, it has been previously suggested that humans are innately prepared to defend themselves against the visual cues of predators (Ohman and Mineka, 2001) and, given that one of the major functions of the human olfactory system has been proposed to be threat detection based on evolutionary experience (Stevenson, 2010), we may likewise have preparedness toward their odors. In humans, a potent analog of a sulfur-based predator odor [2-methyl-2-thiazoline (2MT)] (Kobayakawa and Kobayakawa, 2011) has been reported to induce changes in heart rate (R. Kobayakawa, personal communication, September 5, 2007). This odorant has been shown to work on a chemoreceptor in mice (transient receptor potential ankyrin 1; Wang et al., 2018), which is also present in humans. Therefore, in the current study, we used 2MT as a background context while human participants learned fear associations between other stimuli. The aim was to test the hypothesis that human fear learning may be modulated by evolutionary ancient olfactory mechanisms.

## Materials and Methods for Main Experiment

### Ethics Statement

This study was approved by the Ethics Committee of the Review Board of Advanced Telecommunications Research Institute International, Japan, and all experiments were performed in accordance with relevant guidelines and regulations. All participants provided written informed consent prior to participation.

### Participants

Participants were 40 Japanese females with normal or corrected to normal vision. Only female participants were recruited to exclude the potentially confounding factor of sex on conditioning (Dalla and Shors, 2009; Inslicht et al., 2013) and to reliably measure skin conductance, which is often found to be more reactive among females (Eisdorfer et al., 1980; Chentsova-Dutton and Tsai, 2007). All participants were right-handed. Each participant was paid ¥5,000 per day for 5 consecutive days, with a bonus ¥5,000 on the last day (¥30,000 in total). Participants were randomly assigned to complete conditioning in the 2MT context (2MT group) or in the control odor context (Control group). The data of four participants (two from each group) were excluded from all analyses. This was due to technical issues with the skin conductance response (SCR) electrodes during the Test phase of the experiment, which was the phase of most interest. There remained 18 participants in each group. There was no significant difference in age between groups [for the 2MT group: *M* = 26.5 years, *SD* = 4.4; for the Control group: *M* = 25.6 years, *SD* = 4.1; *t*(34) = 0.63, *p* = 0.532].

The considerable time required to run each participant in the main experiment (1.5 h per day × 5 days per participant) necessitated a modest sample size (20 participants per group × 2 groups). A simple *post hoc* power analysis [for a between-group independent-samples *t*-test examining average SCRs to CS+ s (minus those to the CS−) from the Test phase of the main experiment] revealed relatively high power (1- β error probability = 0.96). We therefore believe that the sample size used in the main experiment was sufficient and appropriate for our experimental design and hypotheses.

### Materials

#### Stimulus Presentation

Visual stimuli were presented on an LCD display using the psychophysics toolbox (Brainard, 1997) running on Matlab 7.1 (MathWorks, Inc.).

#### Physiological Measurements and Electric Stimulation

SCR was recorded *via* BrainVisionRecorder using BrainAmp (Brain Products GmbH, Germany) Ag/AgCl sintered electrodes. Electric shocks were administered *via* electrodes that were connected to a Digitimer constant current stimulator (DS7A-SP; Digitimer, Welwyn Garden City, United Kingdom). The SCR recording electrodes were attached to participants’ index and middle fingers on their non-dominant hand. The electric shock electrodes were attached to the forearm of each participant’s dominant hand. Participants completed all phases of the experiment with their head in a headrest, with the aim of reducing their body movement and therefore movement-related SCR artifacts.

#### Conditioned and Unconditioned Stimuli

We selected three male angry face photos from the ATR Facial Expression Database (DB99; ATR-Promotions, Kyoto, Japan)^1^ to be used as CS. We selected these stimuli because it has been previously shown that fear responses are readily acquired to angry face stimuli (Esteves et al., 1994) and that anger is more easily perceived in male than in female faces (Becker et al., 2007). For each participant, one of these photos was delegated as her “Main CS+” (for which she did not undergo extinction training), a second as her “Extinction CS+” (for which she did undergo extinction training), and the third as her “CS−.” These were counterbalanced between participants. The US used was electric shock, the strength of which was individually adjusted for each participant to a level that she reported as uncomfortable but not painful. For each instance of US presentation, the participants experienced a series of five shocks over 50 ms. The average output current was 32.50 mA (*SD* = 8.30 mA) from a source voltage of 200 V. The pulse width range was 50 μs. There was no difference in output current between the two groups of participants in either the Acquisition or the Test phases (*p*s > 0.05).

#### Odors

In order to determine which odors to use for each individual, prior to experimentation, we had each participant rate seven odors for familiarity, pleasantness, and how much she liked them on a scale from −5 to + 5. Although “pleasantness” and “like” may appear close to equivalent in English, these ratings were completed in Japanese, where the question for “pleasantness” conveyed a meaning akin to questioning how objectively pleasant the participants thought the odors were and the question for “like” conveyed a meaning akin to questioning how much they subjectively liked the odors. The rationale for including both of these was that we considered that some people might consider themselves to like (/dislike) a given odor, even while they think that in general most people would dislike (/like) it. The odorants used to make these odors were: 2MT, Jalapeño brine, konjac brine, Japanese pickled ginger brine, black bean paste, rooibos tea, and Seirogan^TM^ (a Japanese herbal medicine). For all odorants other than 2MT, 1.00 μl was applied to a cotton pad that was presented in a jar for rating. For 2MT, a smaller amount was advised by its designer (R. Kobayakawa, personal communication, April 6, 2016), and so 0.10 μl was applied to a cotton pad that was presented in a jar for rating. Using this smaller amount of 2MT allowed for subjective ratings to this which were comparable to those given to the other odorants (see below). Throughout experimentation, the odors were “contextually” presented in facial masks. Specifically, for each participant for each “odor context,” the relevant odorant was applied to two cotton pads. Half the amount presented from the odor rating phase was applied to each cotton pad so that in total the participants received the same concentration of the odorants as they had experienced during the rating phase. One of these cotton pads was inserted into the left- and the other into the right-sided pocket of the facial mask, which the participant subsequently wore.

During the Acquisition phase, all participants in the 2MT group were presented contextually with the predator odor 2MT (Kobayakawa and Kobayakawa, 2011). Each participant in the Control group was presented contextually with the odor that she had rated most similarly to 2MT. For both groups, odors presented during the Acquisition phase shall be referred to as “acquisition odors.” Overall for the Control group, the following acquisition odors were used: konjac brine for six participants, Seirogan^TM^ for five participants, black bean paste for three participants, Japanese pickled ginger brine for three participants, and Jalapeño brine for one participant. Odors were also presented contextually during the Extinction phase of this paradigm (“extinction odors”). For each participant, this was the odor that she had rated most similarly to her acquisition odor. The exception was that, despite being rated similarly to their acquisition odor, 2MT was never used as the extinction odor for the control group. Overall, the following extinction odors were used: Japanese pickled ginger brine for 12 participants, Seirogan^TM^ for seven participants, Jalapeño brine for six participants, konjac brine for five participants, black bean paste for five participants, and roibos tea for one participant. Each participant’s acquisition odor was presented contextually during one session of the Test phase, and her extinction odor was presented contextually in the other. This order was counterbalanced between participants.

Participants’ ratings to their acquisition and extinction odors are shown in Supplementary Figure S1. For each type of rating (familiarity, pleasantness, and liking), a Group (2MT or Control) by Odor (their acquisition or extinction odor) repeated-measures analysis of variance (ANOVA) was conducted. The results showed that, overall, the acquisition odors were rated as less pleasant [*F*(1, 34) = 8.22, *p* = 0.007], less familiar [*F*(1, 34) = 16.56, *p* < 0.001], and less liked [*F*(1, 34) = 9.42, *p* = 0.004] than the extinction odors. These effects of Odor did not interact with Group for pleasantness [*F*(1, 34) = 0.26, *p* = 0.616] or for liking [*F*(1, 34) = 0.03, *p* = 0.866]. An interaction between Odor and Group was found for familiarity [*F*(1, 34) = 4.31, *p* = 0.046]. Importantly, however, follow-up independent-sample *t*-tests showed no significant between-group differences for familiarity ratings of the acquisition odors [*t*(34) = −1.61, *p* = 0.117] or for familiarity ratings of the extinction odors [*t*(34) = −0.11, *p* = 0.915]. Instead, and of no major consequence for our experimental results, follow-up paired-sample *t*-tests showed that the 2MT [*t*(17) = −3.95, *p* = 0.001], but not the Control group [*t*(17) = −1.59, *p* = 0.131], rated their acquisition odor as less familiar than their extinction odor.

### Experimental Procedure

#### Acquisition Phase (Day 1)

**FIGURE 1:**
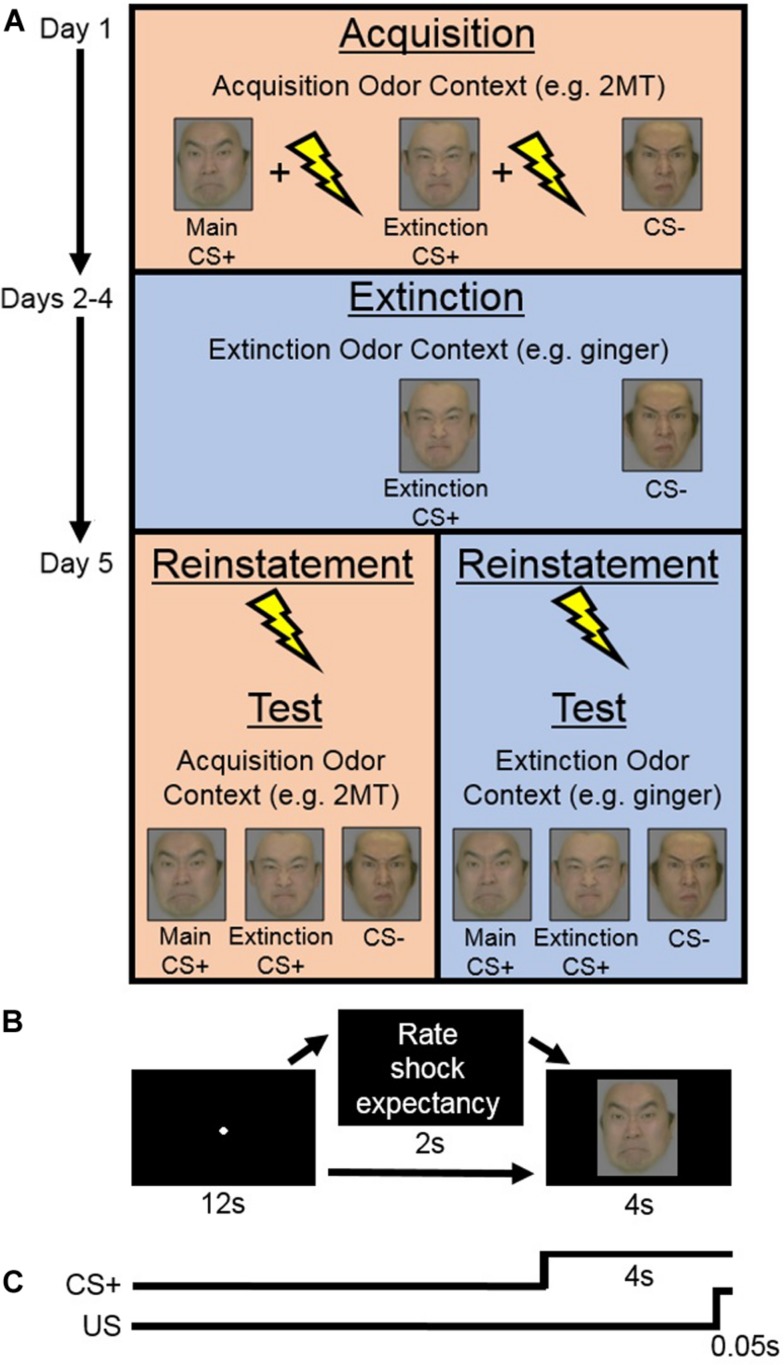
**(A)** Experimental design of the main experiment. On Day 1 (Acquisition phase), in her Acquisition Odor Context [2-methyl-2-thiazoline (2MT) or control odor], each participant learned to associate both conditioned stimuli (CS)+ s, but not the CS−, with the unconditioned stimuli (US). Next, on Days 2–4 (Extinction phase), in her Extinction Odor Context, each participant was exposed to multiple presentations of the Extinction CS+ and the CS−, with no US presentation. Finally, on Day 5, in the two sessions of the Test phase, each participant was presented with all the CS. Reinstatement with the US was completed at the beginning of each Test session. One of these sessions was completed in the participant’s Acquisition Odor Context and the other in her Extinction Odor Context. This order was counterbalanced between participants. **(B)** On each trial of the Acquisition and Test phases, participants saw a central fixation point for 12 s. This was followed in some cases by a screen that appeared for 2 s with instructions for participants to rate electric shock (US) expectancy once the CS appeared on screen. Finally, a CS was presented for 4 s. **(C)** On most CS+ trials during the Acquisition phase, the US was presented during the last 0.05 s of CS+ presentation.

The Acquisition phase consisted of two sessions. During each session, both of the CS+ s (Main CS+ and Extinction CS+) were presented in nine trials each accompanied by the US and in another four trials each without the US. This intermittent CS+ -US schedule was selected, in line with previous human fear-conditioning studies (e.g., Norrholm et al., 2006; Schiller et al., 2008), with the hope that it would help to prevent participants from habituating to the US (as was suggested by Sehlmeyer et al., 2009). The CS− was presented in nine trials without the US. On 10 out of the total 35 trials per session, participants were prompted to rate their expectation of receiving electric shock. Specifically, this rating was prompted on two trials for each CS+ with US, for two trials for each CS+ without US, and on two trials for the CS− (see Figure 1A).

Within each trial (Figure 1B), a screen with a central fixation point was presented for 12 s followed by a screen displaying a CS for 4 s. On US trials, the US was presented during the last 0.05 s of the CS presentation (Figure 1C). On the trials where participants were prompted to rate US expectancy, the following question was presented for 2 s in between the fixation and the CS: “Do you think you will get an electric shock on the next trial? Please reply as soon as possible after the face image is presented.” Then, once the CS appeared on screen, participants responded whether they thought they would “definitely get a shock,” “probably get a shock,” “probably not get a shock,” or “definitely not get a shock” with their dominant hand using the computer keyboard. Response key assignments were counterbalanced between participants. The trial order was randomized within each session. Each participant completed the Acquisition phase wearing a facial mask containing cotton pads sprayed with her acquisition odor. Note that, even if unpleasant, these odors should not act, themselves, as US to be specifically associated with the CS+ s. This is because each participant’s “odor mask” was worn throughout her entire Acquisition phase and therefore it is unlikely that she would have learned to associate the odor any more with the CS+ s than with the CS−. SCR data were recorded throughout.

#### Extinction Phase (Days 2–4)

Participants completed three extinction sessions per day for three consecutive days to see if any effects found were robust enough to survive extensive extinction training (Figure 1A). In each session, they were presented with the Extinction CS+ and the CS− 15 times each in a randomized order. The US was not presented on any trials. Within each trial, participants saw a central fixation point on screen for 7 s, followed by a CS for 6 s, and then a blank screen for 7 s. Each participant completed the Extinction phase wearing a facial mask containing cotton pads sprayed with her extinction odor. SCR was recorded throughout.

#### Reinstatement and Test Phase (Day 5)

Prior to the Test phase, the strength of electric shock was individually readjusted for each participant to a level that she considered to be uncomfortable but not painful. There were two sessions of the Test phase (Figure 1A), each of which was preceded by a reinstatement procedure (Bouton, 2004). During reinstatement, over the course of 1 s, participants were given four series of five unsignaled electric shocks (one series every 250 ms), while being presented with a blank screen. The blank screen then remained on for 10 more min, during which participants were instructed to simply relax, after which the Test session started.

Within each of the two sessions of the Test phase, participants saw each of the three CSs seven times (21 trials in total). On two trials for each CS, participants were prompted to rate their US expectancy in the same manner as in the Acquisition phase. Trials were presented in a random order, except that the CS− was always presented on the first trial (to capture the orienting effect; Bouton, 2004; Koizumi et al., 2016), and the Main and Extinction CS+ s were always presented second and third in random order. Because SCR is often found to be most prominent in the first few trials of testing, we kept the US expectancy rating prompt out of these trials to prioritize SCR measurement. The sequence of events within Test phase trials was identical to those within Acquisition phase trials except that the US was never presented (Figure 1B). During the Test phase, each participant wore a facial mask containing cotton pads sprayed with her acquisition odor in one session and her extinction odor in the other. This order was counterbalanced between participants. The SCR was recorded throughout.

### Statistical Analyses

#### Skin Conductance Responses

SCRs for each phase of this experiment (Acquisition, Extinction, and Test) were analyzed with the Matlab analysis software Ledalab V3.4.9^2^. The data from each participant were down sampled to 10 Hz and manually artifact corrected for each phase (Boucsein et al., 2012). Then, optimized continuous deconvolution analysis (Benedek and Kaernbach, 2010) was performed. This analysis involves deconvolution of skin conductance data by the general physiological SCR shape to separately extract continuous tonic and phasic drivers of skin conductance (Benedek and Kaernbach, 2010). SCR data from the phasic driver were standardized using a z-score transformation. The SCR amplitude for each trial for each participant was calculated by subtracting the trough from the peak data point within the 1–4 s time window after CS onset. The data were normalized using a log transformation (Braithwaite et al., 2015). The processed data were averaged separately for each participant for each CS.

#### Unconditioned Stimulus Expectancy

Participants’ ratings that they thought they would “definitely get a shock,” “probably get a shock,” “probably not get a shock,” or “definitely not get a shock” were coded as ratings of 3, 2, 1, and 0, respectively. The averages of these were then taken in each relevant phase to determine their US expectancy.

## Results of the Main Experiment

### Skin Conductance Responses in the Acquisition Phase

**FIGURE 2:**
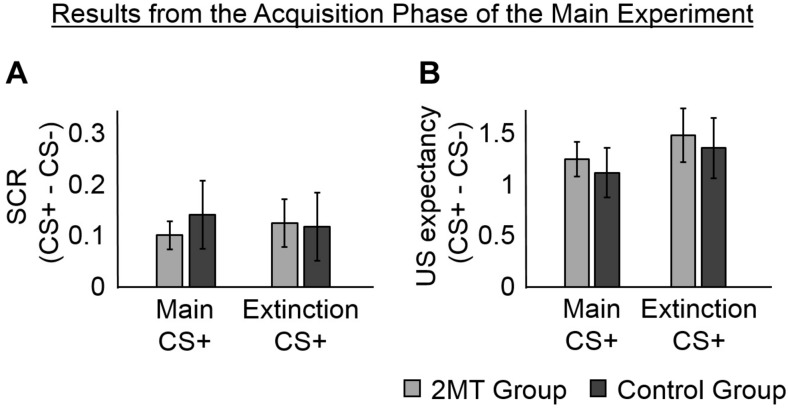
Results of the Acquisition phase. Skin conductance responses (SCRs) (peak-trough) were z-scored, log-transformed, and averaged across participants in each group. Unconditioned stimuli (US) expectancy ratings (from 0 to 3) were averaged across participants in each group. Overall, participants in both the 2-methyl-2-thiazoline (2MT) and Control groups were similarly successful in fear acquisition. For both SCR and US expectancy, mean responses to the conditioned stimuli (CS)- were subtracted from those to the CS+ s to display fear-specific effects. Error bars represent standard error of the mean. **(A)** The SCRs of both groups, were significantly greater to the two CS+ s than to the CS−. This provides evidence for successful acquisition of CS−US associations. **(B)** Likewise, the US expectancy ratings of both groups, were significantly greater to the two CS+ s than to the CS.

To ensure that participants learned to associate the CS+ (but not the CS−) with the US, we examined the SCR in the Acquisition phase that occurred in the 1–4 s window after each CS onset (Figure 2A). The SCR in this time window is expected to be untainted by potential responses to the US, despite the US being presented 3.95 s after the CS in some trials. This is because the onset of the SCR is estimated to arrive at the sweat glands of the fingers about 1.6 s (± 0.03) after electrical stimulation (Lim et al., 2003).

Two linear mixed-effects (LMEs) models were estimated and compared to determine whether or not including a random intercept of subject helped to better explain the data. The mean SCRs (for each participant to each CS) were the dependent factors in both models. The first model had fixed effects of Group (2MT or Control group), CS (Main CS+, Extinction CS+, or CS−), and of the interaction between Group and CS. The second model had the same fixed effects and a random intercept of subject. Data from 3 of the 36 participants (2 from the 2MT group) was excluded from these models because of technical issues with skin conductance electrode during the Acquisition phase. Because the US expectancy ratings of these 3 participants showed they could clearly discriminate between the CS+ s and the CS− (Supplementary Information), and because there were no technical issues with the skin conductance electrodes for these participants during the Test phase (which was of most interest), their data were included in all other analyses. A likelihood ratio test showed that the second model, which included a random intercept of subject, explained SCR significantly better [Akaike’s Information Criterion (AIC) = −20.27] than did the first model [AIC = 32.15; χ^2^(1) = 54.41, *p* < 0.001]. This, and all subsequent likelihood ratio tests, were computed using the Statistics and Machine Learning Toolbox^TM^ for Matlab.

An ANOVA for the fixed effects of the second model showed a significant main effect of CS [*F*(2, 93) = 3.57, *p* = 0.032] (this and all other ANOVAs of LME models were calculated using the Statistics and Machine Learning Toolbox^TM^ for Matlab). No significant effect of Group [*F*(1, 93) = 0.85, *p* = 0.358] and no significant Group by CS interaction [*F*(2, 93) = 0.28, *p* = 0.759] were found, indicating that physiological responses did not differ between groups in the Acquisition phase. Consistent with proper acquisition of CS−US associations, a follow-up *t*-test showed that SCRs were significantly greater to the Main CS+ [*M* = 0.67, *SD* = 0.23; *t*(32) = 3.44, *p* = 0.002] and to the Extinction CS+ [*M* = 0.67, *SD* = 0.30; *t*(32) = 3.06, *p* = 0.004] than they were to the CS− (*M* = 0.55, *SD* = 0.28). There was no significant difference in SCRs to the two CS+ s [*t*(32) = 0.03, *p* = 0.973].

### Unconditioned Stimulus Expectancy in the Acquisition Phase

To further ensure that participants had learned to associate the CS+ s (but not the CS−) with the US, we examined the ratings that they gave in some trials of the Acquisition phase about how likely they thought they were to receive the US. The same two LME models that were used to analyze SCRs in the Acquisition phase were run again, but with mean US expectancy ratings from the Acquisition phase (Figure 2B) as the dependent factor. There was no significant difference between the models [χ^2^(1) = 0, *p* = 1.000; AIC = 210.91 for the first model, AIC = 212.91 for the second model]. An ANOVA of the first model (which only had the fixed effects) showed a significant main effect of CS [*F*(2, 102) = 33.17, *p* < 0.001]. No significant main effect of Group [*F*(1, 102) = 0.02, *p* = 0.890] and no significant Group by CS interaction [*F*(2, 102) = 0.10, *p* = 0.902] were found, indicating that US expectancy was equal for both groups of participants.

In evidence that participants had gained a proper understanding of CS−US associations, follow-up *t*-tests showed that mean US expectancy ratings were significantly greater to the Main CS+ [*M* = 2.29, *SD* = 0.65; *t*(35) = 7.58, *p* < 0.001] and the Extinction CS+ [*M* = 2.08, *SD* = 0.50; *t*(35) = 8.45, *p* < 0.001] than they were to the CS− (*M* = 0.83, *SD* = 0.68). There was no significant difference in mean US expectancy ratings between the two CS+ s [*t*(35) = 1.80, *p* = 0.081], suggesting that the CS−US associations were similarly learned for these.

### Skin Conductance Responses in the Test Phase

**FIGURE 3:**
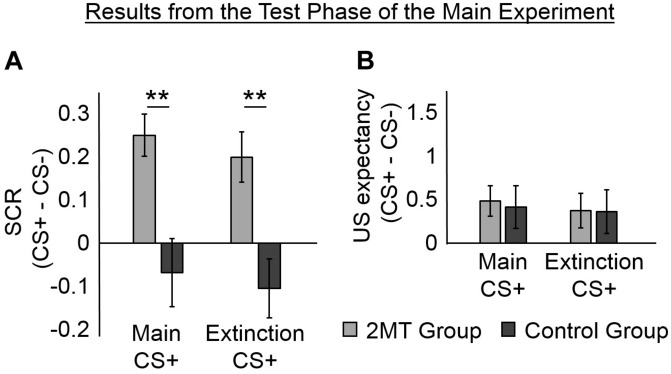
Results of the Test phase. Skin conductance responses (SCRs) (peak–trough) were z-scored, log-transformed, and averaged across participants in each group. Unconditioned stimuli (US) expectancy ratings (from 0 to 3) were averaged across participants in each group. Mean responses to the conditioned stimuli (CS)- were subtracted from those to the CS+ s to display fear-specific effects. Furthermore, because no effects of the odor context used in the Test phase were found, mean responses were averaged across these for ease of display. Error bars represent standard error of the mean. **(A)** Overall, for the 2-methyl-2-thiazoline (2MT) group, SCRs were significantly stronger to the two CS+ s than to the CS−. No significant differences were found for the Control group. For demonstrative purposes, independent-sample *t*-tests were conducted to compare (CS+ – CS−) between groups (collapsed across the two Odor Contexts used in the Test phase). Both showed significant differences (**p < 0.01). **(B)** On average, both groups rated US expectancy as higher on the CS+ than on the CS− trials, with no apparent difference between the groups.

We examined SCRs from the Test phase that occurred four days after the Acquisition phase to see if participants still had greater SCRs to the CS+ s than to the CS− (Figure 3A and Supplementary Figure S2). Each participant completed two sessions of the Test phase: one in her Acquisition Odor Context and one in her Extinction Odor Context (the order was counterbalanced between participants).

We ran two LME models with mean SCRs from the Test phase as the dependent factor. The first model had fixed effects of Group (2MT or Control group), CS (Main CS+, Extinction CS+, or CS−), Odor Context (Acquisition or Extinction Odor Context), and of the interactions between Group, CS, and Odor Context. The second model had the same fixed effects and a random intercept of subject. *Via* likelihood ratio testing, the second model (AIC = 194.55) was shown to better explain mean SCRs in the Test phase than the first model [χ^2^(1) = 185.82, *p* < 0.001; AIC = 378.37].

An ANOVA of the fixed effects of the second model showed a significant main effect of CS [*F*(2, 204) = 5.09, *p* = 0.007]. This was qualified by a significant CS by Group interaction [*F*(2, 204) = 3.31, *p* = 0.038]. There was neither a significant main effect of Odor Context [*F*(1, 204) = 0.16, *p* = 0.692] nor any significant interactions involving Odor Context [for its interaction with CS: *F*(2, 204) = 0.37, *p* = 0.691; for its interaction with Group: *F*(1, 204) = 0.29, *p* = 0.590; for the three-way interaction between Odor Context, CS, and Group: *F*(2, 204) = 0.22, *p* = 0.803].

Follow-up paired sample *t*-tests (collapsed for Odor Context) were conducted using a Bonferroni adjusted alpha level of 0.008 (0.05/6). The 2MT group were found to have significantly higher SCRs to the Main CS+ [*M* = 0.83, *SD* = 0.47; *t*(17) = 5.14, *p* < 0.001] and to the Extinction CS+ [*M* = 0.78, *SD* = 0.44; *t*(17) = 3.42, *p* = 0.003] than to the CS− (*M* = 0.58, *SD* = 0.32). They showed no significant difference in SCRs to the two CS+ [*t*(17) = 0.83, *p* = 0.421]. These results indicate that the 2MT group still displayed fear-like responses to the stimuli that had been associated with electric shock. In contrast, the Control group did not show significantly larger SCRs to the Main CS+ [*M* = 0.75, *SD* = 0.56; *t*(17) = −0.87, *p* = 0.396] or the Extinction CS+ [*M* = 0.71, *SD* = 0.58; *t*(17) = −1.53, *p* = 0.144] than they did to the CS− (*M* = 0.82, *SD* = 0.67). They also showed no significant difference in SCRs to the two CS+ s [*t*(17) = 0.66, *p* = 0.517].

### Unconditioned Stimulus Expectancy in the Test Phase

We examined whether participants still showed greater US expectancy to the CS+ s than to the CS− in the Test phase (Figure 3B). We ran the same two LME models that were used to analyze SCRs from the Test phase, but with mean US expectancy ratings from the Test phase as the dependent factor. Likelihood ratio testing showed the second model (which had the fixed effects and a random intercept of subject; AIC = 514.19) to better explain US expectancy ratings from the Test phase than the first model [which only had the fixed effects, χ^2^(1) = 90.59, *p* < 0.05; AIC = 602.78]. An ANOVA of the fixed effects of the second model showed a significant main effect of CS [*F*(2, 203) = 3.50, *p* < 0.05]. No main effects of Group [*F*(1, 203) = 0.20, *p* = 0.653] or Odor Context [*F*(1, 203) = 0.00, *p* = 1.000] were found to be significant. Likewise, no significant interactions were found (for the interaction between CS and Context [*F*(2, 203) = 0.05, *p* = 0.949]; for the interaction between CS and Group [*F*(2, 203) = 0.06, *p* = 0.945]; for the interaction between Context and Group [*F*(1, 203) = 0.43, *p* = 0.514]; for the three-way interaction between Context, CS, and Group [*F*(2, 203) = 0.11, *p* = 0.896]).

Because the ANOVA revealed a significant main effect of CS, we conducted follow-up paired sample *t*-tests (collapsed for Group and Odor Context). A Bonferroni adjusted alpha level of 0.008 (0.05/6) was implemented. Overall, participants showed significantly higher US expectancy ratings to the Main CS+ [*M* = 1.11, *SD* = 0.83; *t*(35) = 3.06, *p* = 0.004] than to the CS− (*M* = 0.66, *SD* = 0.92). They also showed higher US expectancy ratings to the Extinction CS+ (*M* = 1.03, *SD* = 0.86) than to the CS− [*t*(35) = 2.33, *p* = 0.025], although this did meet the adjusted alpha level of significance. They showed no difference in US expectancy ratings to the two CS+ s [*t*(35) = 1.06, *p* = 0.295]. These results indicate that both groups of participants explicitly recalled which stimuli had been associated with shock. However, it must be noted that US expectancy ratings were few and were deliberately collected only after the first few trials so as not to interfere with SCR measurement (see section Experimental Procedure). Therefore, we cannot rule out the possibility that between-group or between-context differences occurred early on in each session.

## Materials and Methods for the Follow-Up Cortisol Experiment

Fear memory formation is enhanced by high levels of corticosteroids during learning (Roozendaal and McGaugh, 2011). We therefore speculated that if a 2MT context causes higher levels of corticosteroids in humans, as it has previously been shown to do in mice (Isosaka et al., 2015), then this might explain our above finding of stronger fear memory formation in a 2MT learning context. We conducted a simple follow-up experiment to examine this. Where not else specified, methods and materials were identical to those used in the main experiment.

### Participants

Participants were 22 Japanese females with normal or corrected to normal vision. Each participant was paid ¥6,000. To ensure validity of cortisol measurements, only participants who took no regular medication were recruited. Furthermore, all participants were informed before coming in of a list of requirements. A questionnaire before the start of the experiment ensured that all participants had abided by these. The requirements were as follows: Participants should not drink alcohol or take any medication for 24 h before coming in; they should not smoke or drink anything with caffeine on the day of experimentation; they should wake up before 9 a.m. on the day of experimentation; they should not eat anything, drink anything but water, or exercise for 2 h before coming in; they should not brush their teeth for 1 h before coming in. Participants were randomly assigned to the 2MT context (2MT group) or the control odor context (Control group). The data of two participants were excluded from the analyses. The data of one participant were excluded because she had extremely high baseline saliva levels that were above the typical range for her age-group (Aardal and Holm, 1995). The data of the other were excluded because, unlike all other participants, she rated all the odors extremely positively. Of the remaining 20 participants, 10 were in the 2MT group and 10 were in the Control group. There was no significant difference in age between groups [for the 2MT group: *M* = 24.8, *SD* = 4.7; for the Control group: *M* = 24.2, *SD* = 4.9; *t*(18) = −0.28, *p* = 0.781].

### Salivary Cortisol Collection and Analysis

Participants provided each saliva sample *via* the Salimetrics passive drool method (Salimetrics LLC, State College, PA, United States) with the aim of providing at least 1.5 ml for each sample. Samples were stored at −80°C, then thawed and centrifuged at 1,500 × g for 15 min at 4°C to remove mucins and other particulate matter. Clear saliva samples were aliquoted into new sample tubes and stored at −80°C until the day of a cortisol assay. The saliva samples were assayed for cortisol using an enzyme-linked immunosorbent assay and the four-parameter non-linear regression standard curve method recommended by the manufacturer of this assay (Salimetrics LLC, State College, PA, United States). Samples were run in duplicate to ensure reliability. The cortisol data for each participant were standardized, using a square root transformation (Kobayashi and Miyazaki, 2015), before being put into LMEs models.

### Experimental Procedure

Each participant first came in and, after signing a consent form, completed the same odor rating task as was completed in the main experiment. The odor for each participant in the Control group was then selected as that which she rated most similarly to 2MT. These were black bean paste for three participants, Seirogan^TM^ for two participants, konjac brine for two participants, Japanese pickled ginger brine for two participants, and roibos tea for one participant. The odor for all participants in the 2MT group was 2MT. Next, after the participant had completed the questionnaire mentioned above (which also asked for the exact time she woke up and when her last menstrual cycle begun), the SCR electrodes were attached to her. With her head in a headrest in order to reduce motion, the participant then rested while looking at a blank screen for 15 min. After this, her baseline level of salivary cortisol was measured. Subsequently, a facial mask containing an odorant (2MT or control odorant) was put on her, and her level of salivary cortisol was measured 10, 20, and 30 min subsequent to this. All participants kept the facial masks on throughout the experiment except when giving saliva samples. They simply rested in the headrest and looked at a blank screen in between saliva sample collections.

## Results of the Follow-Up Cortisol Experiment

### Questionnaire and Odor Rating Results

Results of the questionnaire showed that factors which could potentially have contributed to salivary cortisol levels did not significantly differ between groups. Specifically, the amount of hours since waking up (for the Control group, *M* = 6.39, *SD* = 2.14; for the 2MT group, *M* = 6.33, *SD* = 1.46) and the amount of days since the beginning of their last menstrual cycle (for the Control group, *M* = 16.10, *SD* = 8.41; for the 2MT group, *M* = 15.60, *SD* = 6.95) were well-matched between groups. Odor ratings were also similar between groups (Supplementary Figure S3). Therefore, these factors are unlikely to have caused any between-group differences in cortisol level.

### Cortisol Results

**FIGURE 4:**
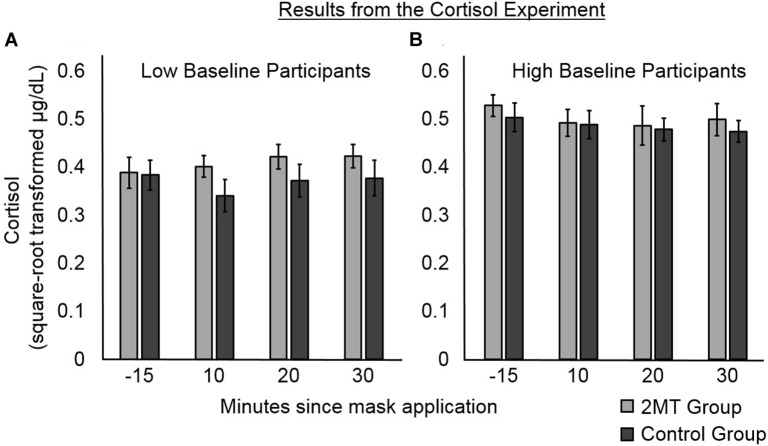
Results of the follow-up cortisol experiment. Error bars represent standard error of the mean. Participants were median split into **(A)** those with lower, and **(B)** those with higher baseline levels of salivary cortisol. For participants with lower baseline levels, post- odor mask application salivary cortisol levels appear higher for those in the 2MT than in the Control group. For participants with higher baseline levels, there were no obvious between-group differences, possibly due to a ceiling effect.

Cortisol data at each Time Point (10, 20, or 30 min after the application of the odor mask), as well as Baseline Level of salivary cortisol (taken 15 min before application of odor masks), were standardized using a square root transformation (Kobayashi and Miyazaki, 2015). Then, two LME models were conducted to analyze the salivary cortisol levels of participants subsequent to application of their odor masks. The dependent variable in each model was standardized cortisol level (Figure 4). The explanatory fixed factors of interest in both models were Group (2MT or Control group), Time Point, and their interaction. As an additional fixed factor in both models, the interaction between standardized Baseline Level of salivary cortisol and Group was also included. This is because despite between-group differences not being significant, Baseline Level of salivary cortisol appeared to differ somewhat between groups, which is of potential concern for such a small sample size. We therefore wished to account for variance potentially explained by between-group differences in this. The first model that we constructed only included these fixed factors. The second included these fixed factors and a random intercept of subject.

Likelihood ratio testing showed the second model (AIC = -229.54) to better explain cortisol levels than the first model [χ^2^(1) = 27.43, *p* < 0.001; AIC = −204.11]. An ANOVA of the fixed effects of this second model showed a significant main effect of Group [*F*(1, 55) = 54.54, *p* < 0.001]. This was qualified by a significant interaction between Group and Baseline Level of salivary cortisol [*F*(1, 55) = 73.19, *p* < 0.001]. No other significant main effects or interactions were found (*p*s > 0.05), indicating that Time Point and the interaction between Time Point and Group did not significantly contribute to the cortisol levels. As can be seen in Figure 4, where participants in both groups have been median split into low and high Baseline cortisol subgroups, there were no apparent between-group differences for participants with high Baseline Levels of cortisol (this may be due to a ceiling effect). However, for the participants with low Baseline Levels of cortisol, cortisol levels subsequent to induction of the odor context appear to be higher for participants in the 2MT than in the Control group. Not surprisingly, given the small sample size for this effect (five participants each for the 2MT/Control low Baseline cortisol subgroups), a follow-up *t*-test examining this effect did not prove significant. The Bayes Factor for this *t*-test was 0.8033 indicating that this (small) data set is not sufficient to support the null or the alternative hypothesis. Nonetheless, the interaction between Group and Baseline Level of cortisol appears of importance because when compared to a model which only had the independent factor of Group (and a random intercept; AIC = −200.76), likelihood ratio testing showed the model that included the interaction (AIC = -229.54) to better explain the data [χ^2^(1) = 30.78, *p* < 0.001].

## Discussion

Our results show that the predator odor 2MT enhances the persistence of physiological aspects of human fear memory associations learned within its context. Participants in this study who learned to associate visual stimuli with electric shock in a 2MT context (2MT group) had greater fear-like SCRs to these visual stimuli in a later Test phase than did participants who learned these associations in a control odor context (Control group). This effect of 2MT appears to be robust because it was found for the 2MT group in both odor contexts of the Test phase, even to a CS+ which had undergone extinction training. The preliminary findings of a follow-up experiment hint that increased levels of cortisol in a 2MT context may provide a potential biological basis for this effect.

Several results indicated that physiological aspects of fear memories formed in the 2MT context were robust. First, fear-like SCRs learned in a 2MT context were strong enough not only to simply last for 3 days (as evidenced in responses to the Main CS+ during the Test phase; Figure 3A) but also to resist 3 days of extensive extinction training (as evidenced in responses to the Extinction CS+ during the Test phase; Figure 3A). Second, the 2MT group displayed fear-like SCRs to the CS+ s even in the Extinction Odor Context of the Test phase (Supplementary Figure S2), further indicating the persistence of their fear responses. This is because, unlike acquisition contexts which are thought to promote retrieval of CS−US associations, extinction contexts are thought to promote retrieval of CS−no US associations (Bouton, 2004). Third, although the preliminary results of a follow-up Long-Term Test (with identical methodology to the main Test; see Supplementary Information) showed that the 2MT group’s SCRs and US expectancy ratings were not significantly different from those of the Control group 3 months later, there was a numeric trend showing them to be higher (Supplementary Figure S4). Overall, the results indicate that the mere presence of a 2MT context during learning strengthened the formation of fear associations.

The corticosteroid levels of mice are increased upon exposure to 2MT (Isosaka et al., 2015), and we therefore hypothesized that the same might be the case for humans. Cortisol is a hormone that is released by the hypothalamus–pituitary–adrenal axis (HPAA) under conditions of high stress/fear; it therefore serves as an endocrine biomarker (Foley and Kirschbaum, 2010). Compared to other biomarkers of stress/fear, cortisol has been particularly linked to memory consolidation and is thus of particular interest for this experiment. Therefore, to investigate whether 2MT affects human levels of cortisol, in a follow-up experiment, we put human participants in either a 2MT or a control odor context and measured their salivary levels of cortisol at several subsequent time points. An interaction between Group (2MT/Control) and baseline levels of cortisol was found to significantly predict salivary cortisol levels after participants were put into their odor contexts. Possibly due to a ceiling effect, this appeared no higher in a 2MT than in a control odor context for participants with high baseline levels. However, for participants with low baseline levels, salivary cortisol appeared higher subsequent to entering a 2MT compared to a control odor context.

Higher cortisol levels in a 2MT context may at least partly explain our results from the main experiment. Previous studies have shown that if the corticosteroid levels of humans and non-human animals are increased during or soon after learning, then this leads to enhanced persistence of memories (Sandi and Rose, 1994; Buchanan and Lovallo, 2001). Furthermore, if this corticosteroid increase is blocked, then this effect is prevented (Roozendaal and McGaugh, 1997). The mechanism behind this effect is thought to be, at least in part (for a detailed description, see Roozendaal and McGaugh, 2011), that corticosteroids bind to receptors in the basolateral amygdala and potentiate the noradrenaline signaling cascade, which in turn enhances memory consolidation processes in other parts of the brain such as the hippocampus. Future studies may directly examine whether a 2MT context enhances fear memory acquisition by engaging these neural mechanisms.

Our participants had probably never experienced 2MT before, and they rated it as unfamiliar, therefore it is unlikely that they had preexisting conditioned threat-relevant associations with this odor. Instead, our results may have arisen because 2MT’s context is one within which humans are “innately prepared” to better learn fear associations. This idea might seem strange, considering that 2MT was derived from the odor of an animal (fox) that does not directly prey upon humans. However, 2MT is sulfur-based (Kobayakawa and Kobayakawa, 2011), and it has previously been proposed that the sulfur metabolites of digested meat indicate the general presence of a potentially dangerous carnivore nearby (Apfelbach et al., 2005). In evidence for this, mice have been shown to make more fear-like responses around the odor of cats who have been fed a carnivorous compared to a vegetarian diet (Berton et al., 1998), and some animals have even been shown to display fear-like responses to the odor of a predator to which their species was previously naive (Mella et al., 2016). Importantly, even humans have been shown to have relatively high sensitivity in detecting sulfur-based predator odors (Sarrafchi et al., 2013). Therefore, although it has previously been speculated that odors gain their affective value for humans *via* learned experience (Herz and Engen, 2010; Li, 2014), our findings suggest that some odors such as 2MT may instead be innately threat-relevant to humans. They raise the questions: in what other scenarios, and to what further extent, might background odors innately modulate human affective and cognitive processes? what other odors might induce similar effects?

In this study, we found that fear-like responses in the Test phase differed depended on whether participants had completed *learning* in the 2MT context but not dependent on whether (for the 2MT group) they were *tested* in the 2MT context. This indicates that 2MT context enhanced the formation, rather than the recall, of fear memories. In the real world, fear memories are often formed in environments that are rich in background olfactory information. Similar to our finding, threat-relevant background odors in the real world may modulate the strength of fear memories formed within their context. One example is that flashbacks in people with post-traumatic stress disorders (PTSDs) are often reported to be triggered by threat-relevant odors that were present in the initial context of a traumatic experience (Vermetten and Bremner, 2003; Hinton et al., 2004). It is possible that, rather than just triggering recall of these memories, these threat-relevant odors may have contributed to the overall strength of memory formation in the first place. Thus, a better understanding of how odor contexts may modulate the strength of fear memory formation may help to elucidate why fear memories are formed so robustly in clinical cases such as PTSDs.

Because the use of a 2MT context during conditioning was found to strengthen physiological aspects of fear memories, this study provides a potential methodological advantage for future researchers. Investigations in human studies are often precluded because the ethically approvable intensity of US (e.g., shock) only leads to weakly formed fear memories. A 2MT context that is subjectively rated to be equally aversive to some other common odors could therefore be utilized in future studies to help strengthen memories of CS−US associations.

It must be noted that in the Test phase of the main experiment, while there was a significant between-group difference in SCRs, there was no significant between-group difference in US expectancy ratings. On average, *all* participants had higher US expectancy ratings to the CS+ s than to the CS− This finding is of potential interest because it fits with the growing literature, suggesting that physiological and self-reported fear memory responses do not always align (Knight et al., 2003) and sometimes can even be dissociated (Bechara et al., 1995). It has been proposed that physical responses to threat might be controlled by a different neural circuit to that which controls the conscious feeling of fear (LeDoux and Pine, 2016; Taschereau-Dumouchel et al., 2019); Our current results may demonstrate the effect of 2MT context specifically on the neural circuit controlling physiological fear memories. However, because of the experimental design used, we cannot rule out the possibility that between-group differences in US expectancy occurred right at the very beginning of the Test phase sessions.

Overall, this study showed that human fear-like responses to visual stimuli that were associated with threat were more persistent when learned in a predator odor context. This may be indicative of “innate preparedness” for fear learning in contexts reminiscent of threats from our evolutionary past. The results of a preliminary follow-up test suggest that this may occur because the predator odor context raises human cortisol levels, which, during learning, is known to lead to stronger memory consolidation. These results indicate that the human olfactory system might have more threat-relevant evolutionary functions that might impact more upon our daily lives than was previously considered. Future experimenters might wish to consider utilizing 2MT contexts as a way to strengthen participants’ CS−US associations. Taken together, our results accord with amassing warnings (Sarafoleanu et al., 2009; Stevenson, 2010) that the human sense of olfaction should not be underestimated.

## Data Availability Statement

The datasets generated for this study are available on request to the corresponding author.

## Ethics Statement

The studies involving human participants were reviewed and approved by Ethics Committee of the Review Board of Advanced Telecommunications Research Institute International, Japan. The participants provided their written informed consent to participate in this study.

## Author Contributions

All of the authors contributed to the experimental design. Data collection and analysis for the Main experiment was conducted by JT. Data collection and analysis for the follow-up cortisol experiment was conducted by JT with much assistance from AN and HS. The manuscript was written by JT and AK, in consultation with and with much advice and input from all other authors. All authors reviewed the manuscript.

## Conflict of Interest

The authors declare that the research was conducted in the absence of any commercial or financial relationships that could be construed as a potential conflict of interest.

## Footnotes

1 http://www.atr-p.com/face-db.html

2 www.ledalab.de

## Supplementary Material

FIGURE S1 Odor-matching in main experiment (potential range = −5 to 5, results are averaged across participants within each group). Overall, there were no significant differences in odor ratings between the 2MT and Control groups. Error bars represent standard error of the mean. These results suggest that any between-group fear memory differences found in this experiment cannot be explained by how pleasant, familiar, and/or how liked the different odors were.

FIGURE S2 Detailed SCR results of the Test phase of the main experiment. SCRs (peak-trough) are z-scored, log-transformed, and averaged across participants in each group. Overall, SCRs of the 2MT group were greater to the two CS+ s than to the CS− in both odor contexts. No such difference was found for the Control group. For demonstrative purposes, for each odor context, paired-sample *t*-tests were conducted within each group to compare SCRs to the different CS (**p* < 0.05; ***p* < 0.01, *****p* < 0.001). Error bars represent standard error of the mean.

FIGURE S3 Odor-matching in Follow-Up Cortisol Experiment (potential range = −5 to 5, results are averaged across participants within each group). Overall, there were no significant differences in odor ratings between the 2MT and Control groups. Error bars represent standard error of the mean.

FIGURE S4 Detailed results of the followup Long-Term Test. SCRs (peak-trough) are z-scored, log-transformed, and averaged across participants in each group. Unlike in the analysis for the results of the original Test, analysis of SCRs in the follow-up Long-Term Test showed no significant effect of CS. Therefore, in this figure, mean responses to the CS− were not subtracted from those to the CS+ s. US expectancy ratings (from 0 to 3) were averaged across participants in each group. Error bars represent standard error of the mean. The top graph shows SCRs, which appear to be higher for the 2MT than for the Control group in both odor contexts. The bottom graph shows US expectancy ratings, which also appear to be higher for the 2MT than for the Control group, except for those to the CS− in the Extinction Odor Context.

FIGURE S5 Results of the Extinction phase. SCRs (peak-trough) are z-scored, log-transformed, and averaged across participants in each group. Mean responses to the CS− were subtracted from those to the CS+ s to display fear-specific effects. Error bars represent standard error of the mean. Overall, neither group showed significant differences between SCRs to the Extinction CS+ and the CS−. This indicates that there was a lack of fear-like responses to the Extinction CS+ during the Extinction phase.
