# Corrigendum: An Evolutionarily Threat-Relevant Odor Strengthens Human Fear Memory

**DOI:** 10.3389/fnins.2020.00638

**Published:** 2020-07-07

**Authors:** Jessica E. Taylor, Hakwan Lau, Ben Seymour, Aya Nakae, Hidenobu Sumioka, Mitsuo Kawato, Ai Koizumi

**Affiliations:** ^1^Department of Decoded Neurofeedback (DecNef), Computational Neuroscience Laboratories, Advanced Telecommunications Research Institute International, Kyoto, Japan; ^2^Department of Psychology, Brain Research Institute, University of California, Los Angeles, Los Angeles, CA, United States; ^3^Department of Psychology, University of Hong Kong, Pokfulam, Hong Kong; ^4^Center for Information and Neural Networks (CiNet), National Institute of Information and Communications Technology (NICT), Osaka, Japan; ^5^Computational and Biological Learning Lab, Department of Engineering, University of Cambridge, Cambridge, United Kingdom; ^6^Department of Neural Computation for Decision-Making, Cognitive Mechanisms Laboratories, Advanced Telecommunications Research Institute International, Kyoto, Japan; ^7^Graduate School of Frontier Biosciences, Osaka University, Osaka, Japan; ^8^Hiroshi Ishiguro Laboratories, Advanced Telecommunications Research Institute International, Kyoto, Japan; ^9^Sony Computer Science Laboratories, Inc., Tokyo, Japan

**Keywords:** fear memory, human olfaction, predator odor, innate fear, contextual memory modulation

In the original article, there was a mistake in the legends for Figures 2 and 3 as published. The ratings should be “(from 0 to 3)” and not “(from −5 to 5)”. The corrected figure legends appear below:

“**Figure 2**. Results of the Acquisition phase. Skin conductance responses (SCRs) (peak-trough) were z-scored, log-transformed, and averaged across participants in each group. Unconditioned stimuli (US) expectancy ratings (from 0 to 3) were averagxed across participants in each group. Overall, participants in both the 2-methyl-2-thiazoline (2MT) and Control groups were similarly successful in fear acquisition. For both SCR and US expectancy, mean responses to the conditioned stimuli (CS)– were subtracted from those to the CS+ s to display fear-specific effects. Error bars represent standard error of the mean. **(A)** The SCRs of both groups, were significantly greater to the two CS+ s than to the CS–. This provides evidence for successful acquisition of CS–US associations. **(B)** Likewise, the US expectancy ratings of both groups, were significantly greater to the two CS+ s than to the CS.”

“**Figure 3**. Results of the Test phase. Skin conductance responses (SCRs) (peak–trough) were z-scored, log-transformed, and averaged across participants in each group. Unconditioned stimuli (US) expectancy ratings (from 0 to 3) were averaged across participants in each group. Mean responses to the conditioned stimuli (CS)– were subtracted from those to the CS+ s to display fear-specific effects. Furthermore, because no effects of the odor context used in the Test phase were found, mean responses were averaged across these for ease of display. Error bars represent standard error of the mean. **(A)** Overall, for the 2-methyl-2-thiazoline (2MT) group, SCRs were significantly stronger to the two CS+ s than to the CS–. No significant differences were found for the Control group. For demonstrative purposes, independent-sample *t*-tests were conducted to compare (CS+ – CS–) between groups (collapsed across the two Odor Contexts used in the Test phase). Both showed significant differences (^**^p < 0.01). **(B)** On average, both groups rated US expectancy as higher on the CS+ than on the CS– trials, with no apparent difference between the groups.”

In addition, the Supplementary Figures are missing legends. The correct legends appear below.

“**Supplementary Figure 1**. Odor-matching in main experiment (potential range = −5 to 5, results are averaged across participants within each group). Overall, there were no significant differences in odor ratings between the 2MT and Control groups. Error bars represent standard error of the mean. These results suggest that any between-group fear memory differences found in this experiment cannot be explained by how pleasant, familiar, and/or how liked the different odors were.”

“**Supplementary Figure 2**. Detailed SCR results of the Test phase of the main experiment. SCRs (peak-trough) are z-scored, log-transformed, and averaged across participants in each group. Overall, SCRs of the 2MT group were greater to the two CS+s than to the CS– in both odor contexts. No such difference was found for the Control group. For demonstrative purposes, for each odor context, paired-sample *t*-tests were conducted within each group to compare SCRs to the different CS (^*^p < 0.05; ^**^p < 0.01, ^***^p < 0.001). Error bars represent standard error of the mean.”

“**Supplementary Figure 3**. Odor-matching in Follow-Up Cortisol Experiment (potential range = −5 to 5, results are averaged across participants within each group). Overall, there were no significant differences in odor ratings between the 2MT and Control groups. Error bars represent standard error of the mean.”

“**Supplementary Figure 4**. Detailed results of the follow-up Long-Term Test. SCRs (peak-trough) are z-scored, log-transformed, and averaged across participants in each group. Unlike in the analysis for the results of the original Test, analysis of SCRs in the follow-up Long-Term Test showed no significant effect of CS. Therefore, in this figure, mean responses to the CS– were not subtracted from those to the CS+s. US expectancy ratings (from 0 to 3) were averaged across participants in each group. Error bars represent standard error of the mean. The top graph shows SCRs, which appear to be higher for the 2MT than for the Control group in both odor contexts. The bottom graph shows US expectancy ratings, which also appear to be higher for the 2MT than for the Control group, except for those to the CS- in the Extinction Odor Context.”

“**Supplementary Figure 5**. Results of the Extinction phase. SCRs (peak-trough) are z-scored, log-transformed, and averaged across participants in each group. Mean responses to the CS– were subtracted from those to the CS+s to display fear-specific effects. Error bars represent standard error of the mean. Overall, neither group showed significant differences between SCRs to the Extinction CS+ and the CS–. This indicates that there was a lack of fear-like responses to the Extinction CS+ during the Extinction phase.”

Finally, a file is missing in the Supplementary Material. It has now been published as Data Sheet 1.

The authors apologize for these errors and state that they do not change the scientific conclusions of the article in any way. The original article has been updated.

