# Supplementary figures and images for: An Evolutionarily Threat-Relevant Odor Strengthens Human Fear Memory
Source: Front Neurosci. 2020 Apr 22;14:255. doi: 10.3389/fnins.2020.00255 (PMC7212458; doi:10.3389/fnins.2020.00255)

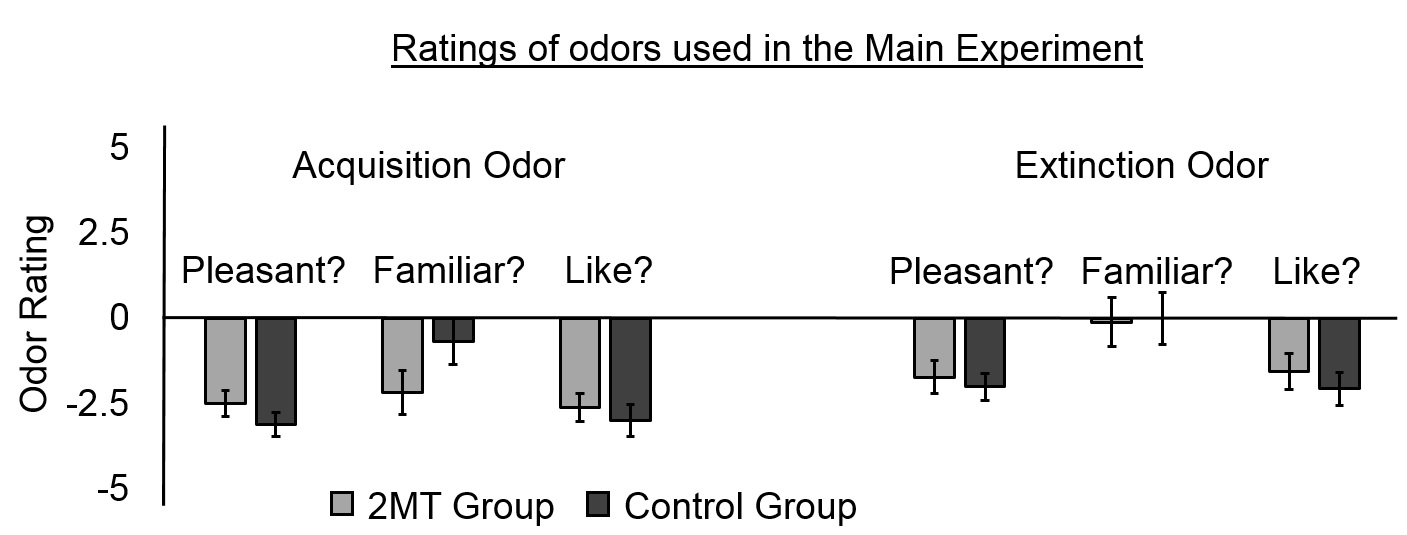

Supplement: FIGURE S1 — Odor-matching in main experiment (potential range = −5 to 5, results are averaged across participants within each group). Overall, there were no significant differences in odor ratings between the 2MT and Control groups. Error bars represent standard error of the mean. These results suggest that any between-group fear memory differences found in this experiment cannot be explained by how pleasant, familiar, and/or how liked the different odors were. [file Image_1.JPEG]

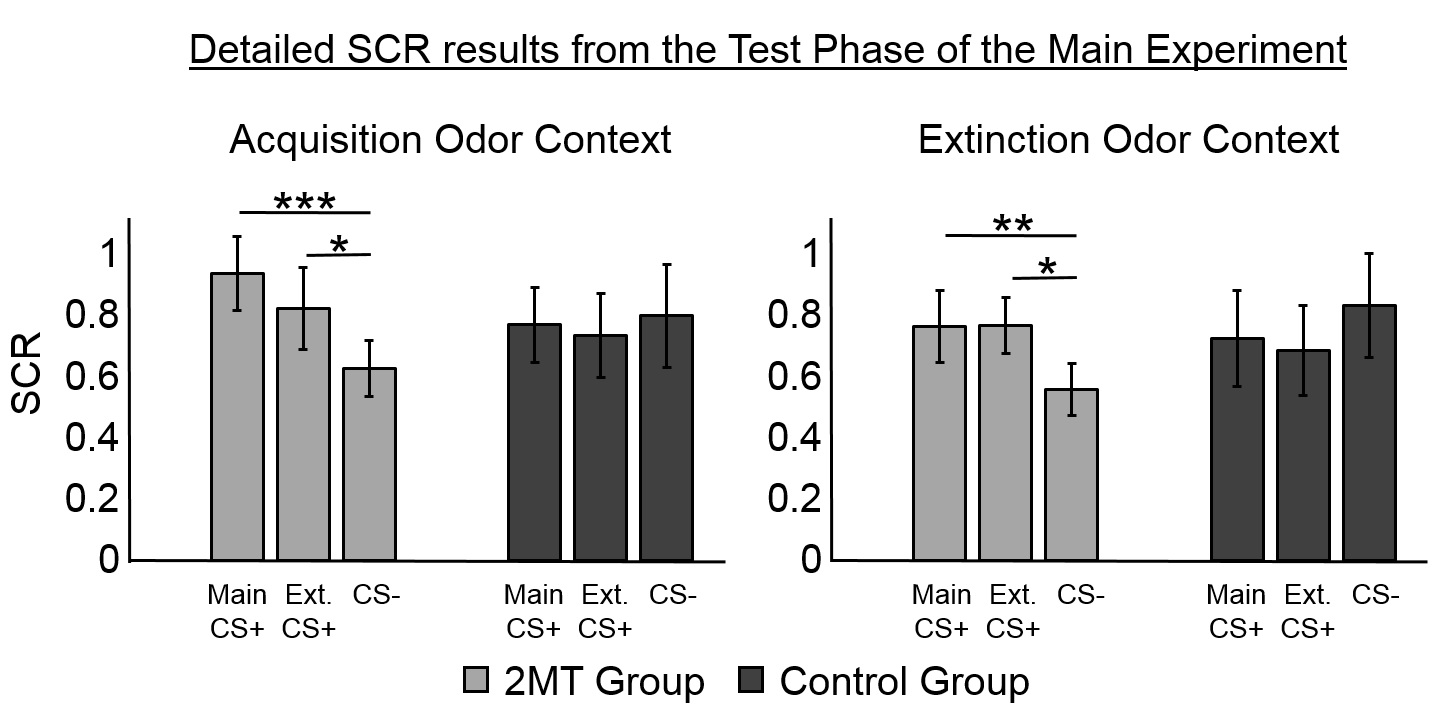

Supplement: FIGURE S2 — Detailed SCR results of the Test phase of the main experiment. SCRs (peak-trough) are z-scored, log-transformed, and averaged across participants in each group. Overall, SCRs of the 2MT group were greater to the two CS+ s than to the CS− in both odor contexts. No such difference was found for the Control group. For demonstrative purposes, for each odor context, paired-sample t-tests were conducted within each group to compare SCRs to the different CS (*p < 0.05; **p < 0.01, ****p < 0.001). Error bars represent standard error of the mean. [file Image_2.JPEG]

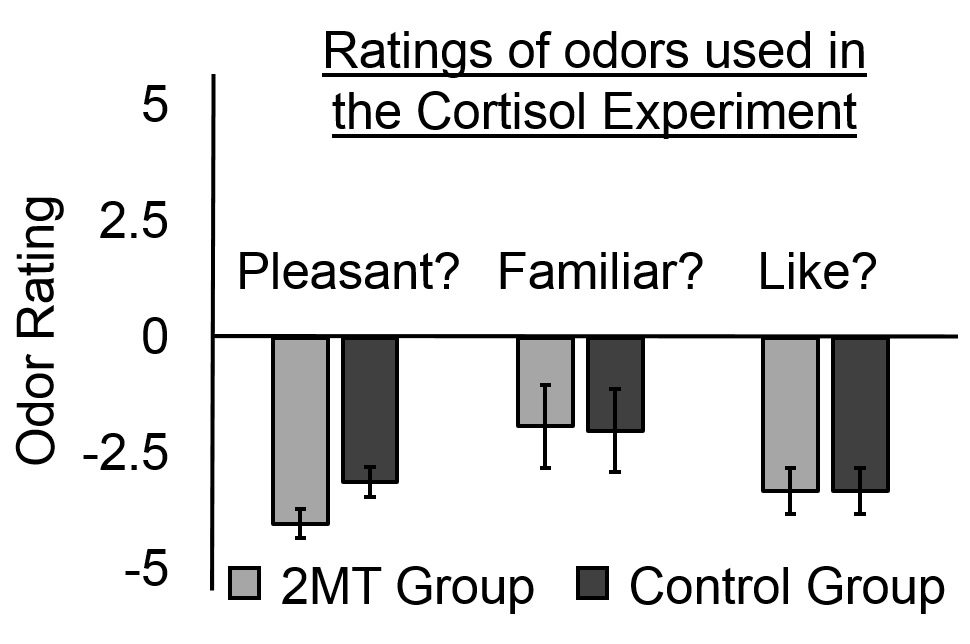

Supplement: FIGURE S3 — Odor-matching in Follow-Up Cortisol Experiment (potential range = −5 to 5, results are averaged across participants within each group). Overall, there were no significant differences in odor ratings between the 2MT and Control groups. Error bars represent standard error of the mean. [file Image_3.JPEG]

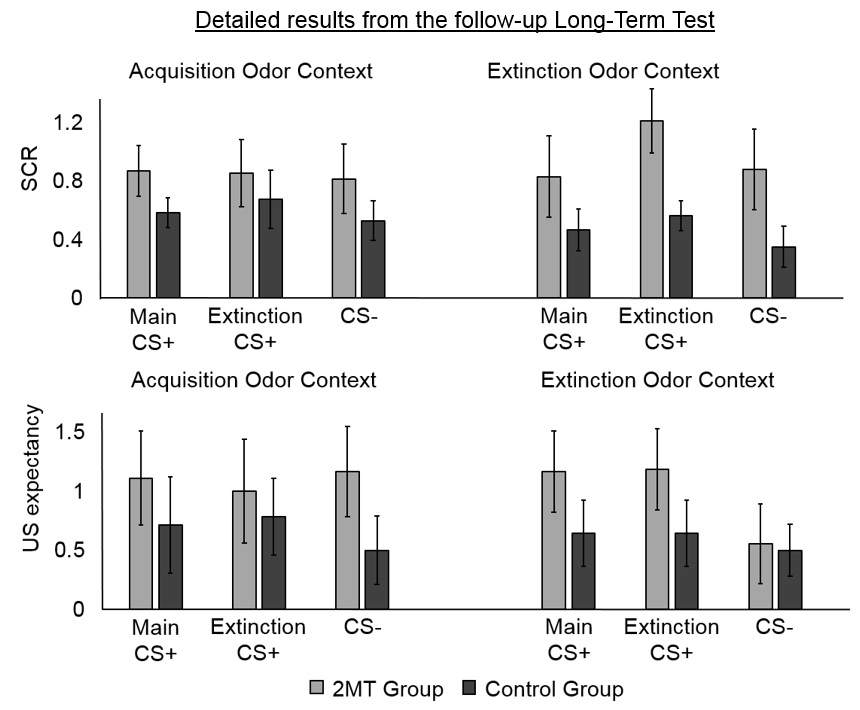

Supplement: FIGURE S4 — Detailed results of the followup Long-Term Test. SCRs (peak-trough) are z-scored, log-transformed, and averaged across participants in each group. Unlike in the analysis for the results of the original Test, analysis of SCRs in the follow-up Long-Term Test showed no significant effect of CS. Therefore, in this figure, mean responses to the CS− were not subtracted from those to the CS+ s. US expectancy ratings (from 0 to 3) were averaged across participants in each group. Error bars represent standard error of the mean. The top graph shows SCRs, which appear to be higher for the 2MT than for the Control group in both odor contexts. The bottom graph shows US expectancy ratings, which also appear to be higher for the 2MT than for the Control group, except for those to the CS− in the Extinction Odor Context. [file Image_4.JPEG]

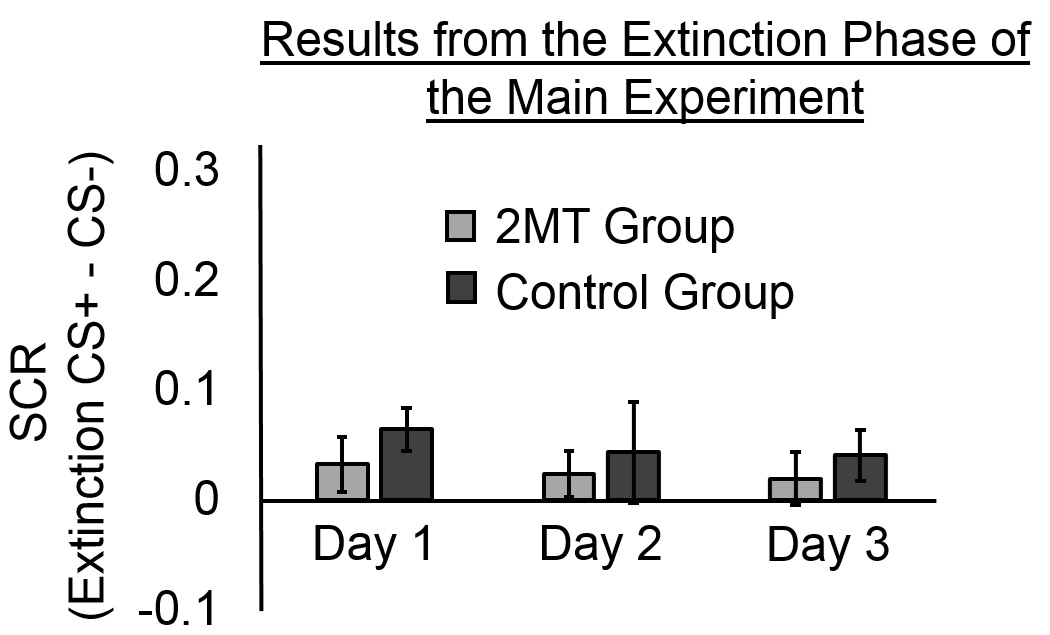

Supplement: FIGURE S5 — Results of the Extinction phase. SCRs (peak-trough) are z-scored, log-transformed, and averaged across participants in each group. Mean responses to the CS− were subtracted from those to the CS+ s to display fear-specific effects. Error bars represent standard error of the mean. Overall, neither group showed significant differences between SCRs to the Extinction CS+ and the CS−. This indicates that there was a lack of fear-like responses to the Extinction CS+ during the Extinction phase. [file Image_5.JPEG]
