# Supplementary material for: An Evolutionarily Threat-Relevant Odor Strengthens Human Fear Memory
Source: Front Neurosci. 2020 Apr 22;14:255. doi: 10.3389/fnins.2020.00255 (PMC7212458; doi:10.3389/fnins.2020.00255)
Supplement: Supplementary file 6 [file Data_Sheet_1.docx]

**Supplementary Information**

**1. Supplementary Results for the Main Experiment**

**1.1 Acquisition Phase US Expectancy for the 3 Participants Excluded from Acquisition Phase SCR Analysis**

The 2 participants from the 2MT group who were excluded from the Acquisition phase SCR analysis had mean US expectancy ratings of 2.63 and 2.13 to the Main CS+, 2.25 and 1.88 to the Extinction CS+, and 0.5 and 1.5 to the CS-. The 1 participant from the Control group who was excluded from the Acquisition phase SCR analysis had mean US expectancy ratings of 2.13 to the Main CS+, 2.75 to the Extinction CS+, and 1.25 to the CS-. Because all of these participants showed higher US expectancy ratings to the two CS+s than to the CS-, they were allowed to proceed to the subsequent phases of the experiment and their data were included in the rest of analyses.

**1.2 SCR in the Extinction Phase**

Because the US was no longer presented alongside the Extinction CS+ during the Extinction phase, SCRs to the Extinction CS+ during this phase were expected to be/become similar to those to the CS- (which was never associated with US). To examine this, two LME models were conducted. Mean SCR from the Extinction phase was the dependent factor. The first model had fixed effects of Group (2MT or Control group), CS (Extinction CS+ or CS-), Day (1, 2, or 3 of the Extinction phase), and of the interactions between Group, CS, and Day. The second model had the same fixed effects and a random intercept of subject. Likelihood ratio testing showed the second model (AIC=166.65) to better explain the SCRs than the first model (χ2(1)=79.21, p<0.001; AIC=243.86). In evidence for a lack of fear responses to the Extinction CS+ during the Extinction phase, an ANOVA of the second model showed no significant main effect of CS (F(1, 200)=0.12, p=0.720). Because no significant main effect of Group was found either (F(1, 200)=2.094, p=0.150), this lack of fear-like physiological response did not depend on the odor context of fear learning. Furthermore, no significant main effect of Day was found (F(2, 200)=1.55, p=0.214) indicating that this lack of fear-like response to the Extinction CS+ occurred right from first day. Further evidence for a lack of differential responses was revealed by the lack of significant interactions between any of the variables (for the interaction between Group and Day: F(2, 200)=1.56, p=0.213; for the interaction between Group and CS: F(1, 200)=0.06, p=0.812; for the interaction between Day and CS: F(2, 200)=1.37, p=0.256; for the 3-way interaction between Group, Day, and CS: F(2, 200)=0.62, p=0.539). See Supplementary Figure 5.

**2. Supplementary SCR Results for the Follow-Up Cortisol Experiment**

SCRs to the five series of unsignalled electric shocks from the end of the cortisol experiment were examined using optimized Continuous Deconvolution Analysis via Ledalab in the same way as in the main experiment. Data was averaged across participants in each group for each series of shocks. The resulting data was then put as dependent variables into LME models. These models were similar to those used to analyze the cortisol data. Group (2MT or Control group), as well as Pleasantness, Time (1st-5th series of shock), and their interaction with Group, were included as explanatory fixed factors in both models. One model only included these fixed effects and the other included these plus a random intercept of subject. No main effects of Group, Pleasantness Rating, or Time, and no interactions were found to be significant in either of the models. This result may indicate that SCRs to the US did not differ dependent on odor context. If true, this would further support the idea that the results of the main experiment indicate that 2MT context affects something about fear memory formation itself, rather than simply affecting initial responses to threatening stimuli.

**3. Supplementary Methods and Results for the Follow-Up Long-Term Test**

**3.1 Methods**

About three months after their original testing, nine participants from the 2MT group and seven participants from the Control group were successfully re-recruited to return for follow-up testing. These participants completed the Test again (with the exact same methodology). The goal was to test whether, even in the long-term, the participants who had acquired fear in the 2MT context had more robust fear memories than the participants who had acquired fear in the control odor context.

**3.2 Results**

The same analyses were run on this data as were run for the original Test, but with mean SCRs and mean US expectancy ratings from the follow-up Long-Term Test as the dependent factors.

Via likelihood ratio testing, the model with fixed effects of Group, CS, Odor Context, and of the interactions between Group, CS, and Odor Context, and with a random intercept of subject (AIC=93.74) was shown to best explain mean SCRs from the follow-up Long-Term Test. This model was significantly better than the model which only had the fixed effects (χ2(1)=78.88, p<0.001; AIC=170.62).

An ANOVA of this model showed a significant main effect of Odor Context; F(1, 81)=8.69, p=0.004. This was qualified by a significant Odor Context by Group interaction; F(1, 81)=6.59, p=0.012. There was neither a significant main effect of CS (F(2, 81)=0.09, p=0.916) nor any interactions involving CS (for the interaction between CS and Context: F(2, 81)=2.50, p=0.088; for the interaction between CS and Group: F(2, 81)=0.20, p=0.816; for the 3-way interaction between CS, Context, and Group: F(2, 81)=1.03, p=0.360).

Follow-up t-tests (collapsed for CS) revealed only one significant result. The SCRs for the Control group were lower in the Extinction Odor Context (M=0.46, S.D.=0.23) than they were in the Acquisition Odor Context (M=0.60, S.D.=0.34; t(6)=2.47, p=0.049). This result fits somewhat with the idea of “return of fear”, which is commonly found when tested in an acquisition context (Bouton, & Bolles, 1979; Bouton, & King, 1983), but not when tested in an extinction context (Bouton, & Bolles, 1979). However, because there was no significant difference in response to CS+s and the CS-, this may be more of a return of context-associated fear rather than a return of stimulus-associated fear. Interestingly, the 2MT group’s SCRs in the Extinction Odor Context (M=0.97, S.D.=0.71) and in the Acquisition Odor Context (M=0.85, S.D.=0.58) did not significantly differ from one another (t(7)=-1.08, p=0.314) and these were both consistently higher than those of the Control group (although not significantly so, perhaps due to the small sample size). Because there was no significant difference in response to CS+s and the CS- for the 2MT group in either odor context, these results may indicate an overall general (i.e. not stimulus-specific) recall of fear that was higher for the 2MT than the Control group. See Supplementary Figure 4

When an analysis of the mean US expectancy ratings from the follow-up Long-Term Test was conducted, the model with fixed effects of Group, CS, Odor Context, their interactions, and a random intercept of subject proved, via likelihood ratio testing (AIC=223.76), to better explain the results than a model with only the fixed effects (AIC=283.51; χ2(1)=61.75, p<0.001). An ANOVA of this model showed a significant main effect of Odor Context; F(1, 83)=5.59, p=0.020. No other main effects or interactions, as well as follow-up t-tests did not reach significance, probably because of the small sample size. However, visual inspection of the data indicates that in the Acquisition Odor Context the 2MT group rated all stimuli as more likely to be associated with US than did the Control group, whereas in the Extinction Odor Context they only rated the CS+s as more likely to be associated with US than did the Control group. See Supplementary Figure 4.

Overall these results provide some preliminary evidence that a 2MT context during learning might strengthen fear memories robustly enough that fear-related effects might still persist in the long-term.

**4. Supplementary References**

- Bouton, M. E. & Bolles, R. C (1979). Role of conditioned contextual stimuli in reinstatement of extinguished fear. J Exp Psychol Anim Behav Process 5(4), 368-378.
- Bouton, M. E. & King, D. A. (1983). Contextual control of the extinction of conditioned fear: tests for the associative value of the context. J Exp Psychol Anim Behav Process 9(3), 248-265.
